# Editorial: Mechanisms of cellular differentiation, organ development, and novel model systems

**DOI:** 10.3389/fcell.2022.970778

**Published:** 2022-08-04

**Authors:** Delilah Hendriks, Benedetta Artegiani, Kai Kretzschmar

**Affiliations:** ^1^ Hubrecht Institute, Royal Netherlands Academy of Arts and Sciences (KNAW) and Oncode Institute, Utrecht, Netherlands; ^2^ Princess Maxima Center (PMC) for Pediatric Oncology, Utrecht, Netherlands; ^3^ Mildred Scheel Early Career Centre (MSNZ) for Cancer Research, University Hospital Würzburg, IZKF/MSNZ, Würzburg, Germany

**Keywords:** stem cells, cellular differentiation, organoids, organ development and homeostasis, disease modelling

Stem cells are undifferentiated cells that can self-renew and potentially differentiate into different specialized cell types. Embryonic stem cells (ESCs) are crucial during development to generate the different organs of the body. In some of the adult tissues, adult stem cells (ASCs) have critical roles for tissue homeostasis and repair upon injury. Understanding the mechanisms that regulate the specific pathways of stem cell differentiation into the different cell types is a central question for stem cell biology. It constitutes the basis to exploit the potential of stem cells for tissue regeneration. Model organisms have been instrumental to investigate stem cell behaviour and differentiation trajectories *in vivo*. In recent years, novel model systems including both 2D and 3D (organoid) cultures have been developed from tissue-resident stem cells (either fetal or adult) and pluripotent stem cells (either embryonic or induced), and those have been particularly critical to leverage our understanding of human stem cells in health and disease. This Research Topic “Mechanisms of Cellular Differentiation, Organ Development, and Novel Model Systems” published in *Frontiers in Cell and Developmental Biology* and *Frontiers in Genetics* explores the recent advancements in stem cell biology with a particular focus on molecular mechanisms driving cellular differentiation in various organs, their dysregulation in a disease context and the optimization and use of novel model systems and techniques.

Although much progress has been made in our comprehension of the mechanisms that drive stem cell renewal and differentiation during embryonic development and later in adulthood in different organs, much remains to be investigated. We need to obtain a more holistic knowledge of the molecular pathways that regulate stem cell behaviour on different levels, including genetic, transcriptomic, and epigenetic changes. Based on this demand, Sevilla et al. used different unbiased approaches including gene-expression and methylation profiling to dissect the molecular changes occurring in mouse embryonic stem cells (mESCs) upon depletion of the key pluripotency regulator estrogen related receptor beta (Esrrb). The study revealed a feed forward loop between Esrrb and the pluripotency factor Nanog that contributes to the timing of mESC differentiation. In embryonic development and adult homeostasis, cell-cell communication is a critical regulator of stem cell fate decisions and lineage selection. Cell-cell communication may be mediated through direct ligand-receptor interactions, cell cytoplasm directly connected via gap junction channels or paracrine signalling enabled, for instance, through pannexin channels that allow passage of signalling molecules out of the cell into the extracellular space. Noort et al. investigated the role of the pannexin channel PANX1 in the lineage specification of human induced pluripotent stem cells (iPSCs). PANX1 is expressed in iPSCs and as well as cell committed to the three germ layers (i.e., ectoderm, mesoderm, and endoderm); however, with distinct patterns of glycosylation and subcellular localization amongst the germ lineages. While loss of PANX1 in iPSCs did not completely elicit the ability of pluripotent cells to differentiate into all germ lineages, it promoted a preferred differentiation towards the endodermal and mesodermal lineages. Undetermined redundancies, interacting partners or signaling molecules contributing to iPSC plasticity may be involved in this process. Ligand-receptor interactions represent a direct form of cell-cell communication and their role in the sex-specific transition from primordial germ cells (PGCs) to gonia during human development was studied by Overeem et al. Focusing on the four major signalling pathways Wnt, Notch, TGF-β/BMP, and receptor tyrosine kinases (RTK), they analysed single-cell transcriptomics data of human fetal gonads (i.e., premeiotic oogonia in females and prospermatogonia in males). The in-silico analysis suggested that sex-specific signalling mimicking the transition from female PGCs to oogonia may rely on activation of Wnt and BMP and further treatment with the RTK ligand KITL. Conversely, activation of RTK signalling by IGF1 and FGF9 treatment and activation of the BMP pathway may promote the transition from PGCs to prospermatogonia in males.

Canonical Wnt signalling is a central pathway regulating (stem) cell lineage selection and differentiation. Its role in promoting the commitment of mesenchymal stromal cells (MSCs) towards adipocytes is reviewed by de Winter and Nusse. Dermal fibroblasts comprise the stromal niche of epidermal stem cells and contribute to their ability to maintain epidermal homeostasis. Both dermal fibroblasts and epidermal keratinocytes are regulated by canonical Wnt signaling. Culley et al. identifed using gene-expression profiling that expression of the Wnt ligand WNT4 and the RTK ligand IGF1 correlates with two distinct human dermal fibroblast lineages. In reconstitution assays of cultured human skin, they showed that IGF1 knockdown increased expression of α-smooth muscle actin (α-SMA), a myofibroblast marker, but did not affect the ability of fibroblasts to support epidermal stratification. Knockdown of WNT4 in cultured dermal fibroblasts, on the other hand, did not alter α-SMA expression, but promoted epidermal stratification. Crosstalk of canonical Wnt with other signalling pathway has been found to be involved in regulating development and maintenance of many different tissues. Hermans et al. summarized the recent literature on the interaction of canonical Wnt and Sonic hedgehog (Shh) signalling during the different stages of murine tooth development, reaching from dental placode formation to the fully formed adult tooth. Integral part of tooth development is the formation of the tooth root that depends on epithelial-mesenchymal interactions. Liu et al. investigated a key process of tooth root development, the lineage selection of stem cells from the apical papilla (SCAP) towards osteo/odontogenic differentiation. Using microRNA (miRNA) microarray analysis, they identified and validated miRNA-497-5p as a positive regulator of osteo/odontogenic differentiation of SCAP. Further bioinformatics analysis and luciferase reporter assays revealed SMAD specific E3 ubiquitin protein ligase 2 (*Smurf2*) is a direct target of miR-497-5p, which suggests that the miRNA promotes osteo/odontogenic differentiation by acting on the Smad signalling pathway. Epithelial-mesenchymal interactions are not only essential during organ development, but they also provide cues underlying tissue homeostasis. In the intestine, stromal cells at the base of the crypts, the niche of intestinal stem cells, produce EGF that drives proliferation of intestinal epithelial cells. Abud et al. reviewed the current knowledge on the cellular sources of EGF family of ligands in the intestine and their impact on intestinal stem cells. They highlight that apart from EGF, members of its ligand family such as Neuregulin 1 play specific roles in maintaining proper intestinal stem cell function by acting through the ErbB-mediated RTK signaling.

The intestine has become a heavily studied model organ to dissect adult stem cell function as well as tissue homeostasis and regeneration**,** due to its rapid turnover. Bonis et al. provided a broad overview of recent findings on the intestinal epithelium and its communication with the underlying mesenchyme. Highlighting how single-cell technologies have led to major discoveries, they discuss how these results have contributed to the better understanding of the maintenance, regeneration, and diseases of the intestine. In comparison to the intestine, the oral mucosa remains a poorly understood tissue. However, the oral mucosa is amongst the few tissues of the human body that can heal with minimal scarring. Pereira and Sequeira discussed this outstanding healing potential by comparing the processes underlying oral mucosal homeostasis and regeneration with those of the skin and esophagus. A better understanding of the mechanisms contributing scarless wound healing in the oral mucosa may open new avenues of research and treatment for achieving the same for scarring tissues such as skin. Ferrario et al. suggested a different approach and make a case for studying invertebrate deuterostomes as models to decipher the cellular aspects of adult regeneration. Invertebrate deuterostomes are a group of animals comprising echinoderms, hemichordates, cephalochordates, and tunicates. Dedifferentiation is discussed as a critical cellular mechanism underlying regeneration in invertebrate deuterostomes, a process that is gaining more or more attention in the field of adult stem cell research, as our understanding of cellular plasticity in adult tissue has increased in the last decade or so.

To obtain a more holistic “-omic” knowledge of the molecular pathways that regulate stem cell behaviour during organ development as well as in adulthood and during disease, it is critical to derive information from the correct platforms of investigation. In recent years, much emphasis has been placed on the optimization and generation of novel model systems, including animal models and *in vitro* (human) stem cell-based cultures, such as organoids. The field of studying the earliest processes underlying embryonic development has seen a considerate increase in development of novel *in vitro* models, including iPSCs, organoids, blastuloids and gastruloids. Such models may bypass some of the ethical concerns associated with culturing human embryos. El Azhar and Sonnen provided an in-depth discussion on how these models are aiding our understanding of pre- and post-implantation development. Blastuloids enable modeling the first steps of pre-implantation, including compartmentalization, lineage segregation, and implantation. Peri- and post-implantation studies are instead aided by the recent establishment of gastruloids, organoids, and so-called “ETX embryos” (ESC, TSC, and XEN cells) to evaluate gastrulation mechanisms, cellular dynamics, and signaling pathways. Development is an extremely delicate and timed process, and defects arising during this process may result, amongst others, in pediatric tumors. Custers et al. and Barbet and Broutier summarized how *in vitro* models aid our understanding of the development of such tumors and how this helps translation to the clinic. Barbet and Broutier further emphasized the importance of both bottom-up and top-down tumor organoids (ie., *tumoroids*). Bottom-up tumoroid models use initially healthy cells as a base to introduce mutations mimicking specific tumor subtypes through genetic engineering approaches, whereas top-down tumoroids represent organoids derived from actual cancer tissue. Both tumoroid types are expected to help unravel the biology of pediatric tumors. The authors highlight the delay in translating tumoroid technology to pediatric tumors, and argue that this could be linked to the origin of the tumor. Whereas pediatric tumors are often of non-epithelial origin (e.g., sarcomas), most cancers in adulthood are derived from epithelial tissues and accordingly establishment of tumoroid cultures have been optimized for epithelial cells. Custers et al. discussed the biological challenges associated with modelling pediatric tumors, related to the notion that mutations likely arise in a specific cell within a specific developmental time window, and thus require extremely controllable and representative *in vitro* models. The authors conclude that although tumor complexity may not be recapitulated in a single model, continued establishment of novel models will collectively fundamentally increase our understanding of pediatric tumors.

Moving on to *in vitro* models of adult tissues, Gähwiler et al. provided a broad review on how iPSCs have been used in cardiac tissue engineering. They discuss how their multiplexing with genome engineering tools, such as CRISPR-Cas9, has contributed to the establishment of robust cardiac disease models. As the heart is an organ with limited regenerative capacity, such engineered cardiac tissues may in the future prove to be valuable in the clinic, but first require addressing safety concerns related to both iPSCs as well as the genome engineering tools. Roos et al. reported a novel culture model that allows to derive cholangiocyte organoids from bile fluid of healthy donors and patients, providing a less-invasive method. Through extensive characterizations, they found that bile-derived organoids are molecularly and functionally very similar to tissue-derived organoids. Interestingly, the organoids were derived under non-canonical Wnt stimulation conditions, which resulted in a better preservation of cholangiocyte-specific basolateral receptor activity as compared to organoids derived under canonical Wnt stimulating conditions.

*In vitro* models, including organoids, also provide a suitable platform to study basic biological processes, such as tissue behaviour in space and time. Betjes et al. discussed the power of cell tracking approaches applied to 3D organoids to answer important questions of organ development and cellular differentiation. While at first glance being a seemingly straightforward concept with the technologies and history in embryo development at hand, they highlight that a current challenge in the field is to find novel ways to efficiently analyse a complex lineage tracking dataset that integrates space, time, lineage, and internal cell states. They argue that combining on the one hand state-of-the-art microscopy approaches and on the other hand state-of-the-art cell visualization techniques will maximize the immense potential of cell tracking to describe organ biology.

In addition to their use for basic biology studies, *in vitro* models equally hold potential for testing clinical therapies. Cappella et al. summarized how iPSCs have been used in the prioritization and testing of therapeutic drug treatments with a focus on neuromuscular and motor neuron disorders. They further discuss the current challenges and selection criteria to identify safe viral vectors for gene therapy and how iPSCs can constitute a valuable testing platform for this purpose.

The use of *in vitro* models is fundamental for the study of human biology, since *in vivo* models are clearly unavailable, and to compare findings to different species for which the use of *in vivo* models is at least controversial and difficult to achieve, such as primates. This type of comparative interspecies studies allows to study cellular and organ functions in an evolutionary perspective, and has been widely applied, for instance, to the study of brain evolution. Within this context, Schörning and Taverna have extensively reviewed the recent studies that made use of PSC-based culture systems, including 3D brain organoids as well as 2D cultures of induced neural populations, to identify evolutionary differences. These include, for instance, diverse mechanisms of specification and maturation of neurons between apes and human. Additionally, this review convincingly illustrates how these advanced culture systems can aid to experimentally test the functional relevance of the multiple genetic differences that exists between humans and their last ancestors, such as Neanderthals and Denisovans. Additionally, PSC-based neural cultures represent powerful tools to understand mechanisms of action and impact on cell identity determination of transcription factors. In this Research Topic, two original studies provide exemplifying cases. Zhang et al. conjugated human retinal organoids with lentiviral-based gene expression manipulation and extensive single cell sequencing analysis to identify the role of two bHLH transcription factors, ATOH and NGN2. The authors found that both transcription factors are instrumental to expand the neuroblasts population and to enhance the production of retinal ganglion cells and cone photoreceptors. In a second example, Cui et al. utilized PSC-derived neural stem cell cultures to manipulate the expression of HIF-1α, and found novel evidence that HIF-1α affects the pluripotency of PSCs and their differentiation capacity by enhancing the expression of Mitofusin2, which ultimately influences pluripotency by regulating the expression of β-catenin.

Beside the importance of these novel and exciting *in vitro* models, the neural stem cell and brain development field has benefitted since long time from *in vivo* model systems, and their great values still holds true, as critical complementary systems. Stepien et al. reviewed for this Research Topic findings obtained from *in vivo* studies as well as integration with human *in vitro* studies, that altogether lead to our current understanding of the human cortical expansion. In particular, the authors focused on interpreting the theoretical and experimental findings showing that the lengthening of the neurogenic period is a key determinant of neocortical expansion, described the genes identified in recent years to affect the neurogenic period length, and discussed the evidence supporting the extrinsic versus the intrinsic regulation of this process. In addition to the more classical and widely used rodent models, mice and rats, different animal models with specific and unique features have showed their importance. Gilardi and Kalebic provided a comprehensive overview of the use of the ferret as an *in vivo* model system to study cortical development and evolution. Specifically, this animal represents an easily accessible and experimentally amenable model, but as compared to others, present with complex cortex folding. As such, it is a particularly appealing system for evolutionary studies. The authors present a clear description of ferret neurogenesis and proceed with highlighting side-by-side commonalities and differences as compared to other species, such as mice and primates (human). In our collection the use of even a more exotic animal model has been documented by Hernández-Núñez et al. in an interesting study aimed at elucidating the signaling pathways that regulate neurogenesis in the adult vertebrate retina: the adult shark. While neurogenesis is retained in specific niches in different vertebrate species, teleost fishes display the most widespread neurogenesis in the adult CNS, however how neurogenesis is conserved in the retina within different species is unclear. Here, the authors present evidence for the persistence of proliferation and neurogenesis in the adult retina of the catshark.

In closing this Research Topic, we believe that this collection provides meaningful examples of how better and novel *in vitro*, as well as *in vivo* models are crucial to advance our understanding of pathways regulating organ development, tissue homeostasis, and how they are involved in disease initiation and progression. Alongside, the studies presented here convincingly show that advancements in state-of-the-art methods and techniques, such as genetic editing, lineage tracing and mathematical modelling, imaging tools, and next generation sequencing including single-cell sequencing, can be increasingly beneficial when applied to the stem cell field.

